# Problems and Opportunities of a Smartphone-Based Care Management Platform: Application of the Wald Principles to a Survey-Based Analysis of Patients’ Perception in a Pilot Center

**DOI:** 10.3390/healthcare12020153

**Published:** 2024-01-09

**Authors:** Stefano Marco Paolo Rossi, Rocco Maria Panzera, Rudy Sangaletti, Luca Andriollo, Laura Giudice, Francesca Lecci, Francesco Benazzo

**Affiliations:** 1 Sezione di Chirurgia Protesica ad Indirizzo Robotico, Unità di Traumatologia dello Sport, U.O.C Ortopedia e Traumatologia, Fondazione Poliambulanza, 25124 Brescia, Italy; roccomaria.panzera01@icatt.it (R.M.P.); luca.andriollo@poliambulanza.it (L.A.);; 2 Università Cattolica del Sacro Cuore, 00168 Roma, Italy; 3 Divisione Government, Health and Not for Profit, CERGAS, SDA Bocconi School of Management (Milano), 20136 Milano, Italy; laura.giudice@unibocconi.it (L.G.); francesca.lecci@unibocconi.it (F.L.); 4 IUSS Istituto Universitario di Studi Superiori, 27100 Pavia, Italy

**Keywords:** care management platform, myMobility, telerehabilitation, robotic knee system, digital ecosystem

## Abstract

(1) Background: Mobile health (mHealth) solutions can become a means of improving functional recovery and reducing the peri-operative burden and costs associated with arthroplasty procedures. The aim of this study is to explore the objectives, functionalities, and outcomes of a platform designed to provide personalized surgical experiences to qualified patients, along with the associated problems and opportunities. (2) Methods: A survey-based analysis was conducted on patients who were prescribed the use of a specific care management platform and underwent primary robotic total knee arthroplasty (rTKA) between January 2021 and February 2023. (3) Results: Patients registered on the platform who have undergone primary robotic TKA (rTKA) were considered. The mean age of registered patients is 68.6 years. The male (M)/female (F) ratio is 45.1%/54.9%. The patients interviewed were at an average distance of 485 days from the intervention, with a standard deviation of 187.5. The survey highlighted appreciation for the app and its features, but also limitations in its use and in its perception by the patients. All these data were evaluated according to the Wald principles and strategies to improve patient recruitment, enhance adherence, and create a comprehensive patient journey for optimized surgical experiences. (4) Conclusions: This patient care platform may have the potential to impact surgical experiences by increasing patient engagement, facilitating remote monitoring, and providing personalized care. There is a need to emphasize the importance of integrating the recruiting process, improving adherence strategies, and creating a comprehensive patient journey within the platform.

## 1. Introduction

In recent years, orthopedic surgery has witnessed a significant evolution in treatment strategies and the way patients approach the post-operative rehabilitation process. This transformation has been driven by several factors, including the increasing adoption of Fast Track protocols and the Enhanced Recovery After Surgery (ERAS) program, aimed at optimizing surgical outcomes and speeding up patients’ healing processes. The expectations of most patients undergoing total knee replacement (TKR) are often unmet [1,2,3], as in some populations, the level of physical activity does not necessarily increase compared to pre-operative levels. As a result, knee prosthetics have undergone a radical transformation, with the focus now not only on the correct execution of the surgical procedure but also on ensuring patients a rapid recovery and a quick return to joint functionality [4,5].

One of the key aspects driving these changes is the desire of patients to regain the efficiency of their knees as quickly as possible. This need, often dictated by factors such as quality of life and the urgency to resume normal daily activities, has led to a thorough reconsideration of the rehabilitation strategies and technologies involved. In this context, studies have been conducted on Mobile Health (mHealth) solutions as a means to improve functional recovery and reduce the peri-operative burden and costs associated with arthroplasty procedures [6].

The widespread adoption of technology by the general public has significantly expanded the scope of mHealth [7,8,9], particularly focusing on the potential of smartphone-based care management platforms (sbCMP) in providing tele-rehabilitation and remote patient monitoring following total joint arthroplasty (TJA) [10,11,12].

Starting in 2017, some smartphones (e.g., iPhone 8, Apple Inc., Cupertino, CA, USA) have been released with a +2 g accelerometer capable of measuring accelerations with output frequencies ranging from 0.5 Hz to 1 kHz (LIS331DLH, STMicroelectronics, Kokomo, IN, USA), a 3-axis gyroscope capable of measuring angular velocity up to 2000 degrees per second (L3G4200D, STMicroelectronics, Kokomo, IN, USA), and a magnetometer (AKM8975, AKM Semiconductor, Tokyo, Japan).

The evolution has also extended to the integration of innovative mobile apps that leverage data collected during surgery, contributing to a more comprehensive and personalized approach to post-operative rehabilitation.

The context of the COVID-19 pandemic and lockdown measures has made the need to reduce travel and hospital visits even more pressing. Limited access to healthcare facilities has emphasized the importance of solutions that allow remote control of the quality and quantity of patient rehabilitation. In this scenario, the need for effective communication between the patient and the care team, without the need for continuous phone calls and outpatient visits, is emerging. Virtual communication is, therefore, an increasingly fundamental component in post-operative care and rehabilitation pathway management [13].

Numerous mHealth systems incorporate wearable devices that gather pre- and post-operative data, which are unaffected by recall bias and can be collected continuously in a passive manner [7,14,15].

Patients undergoing TJA have responded positively to remote therapeutic monitoring platforms that utilize wearables [16,17]. Smartphone-based platforms have shown comparable outcomes to traditional recovery pathways while reducing the need for in-person rehabilitation [10,11,15,18]. These platforms enable the passive collection of mobility metrics, which serve as potential indicators of early functional recovery. While pre- and post-operative mobility data can be valuable for remote monitoring, their correlation with patient-reported outcome measures (PROMs) has yielded mixed results [19,20,21]. Nevertheless, it is necessary to establish more objective and performance-based measures of mobility, as traditional PROMs have limitations due to ceiling effects [22].

The use of apps like myMobility represents a significant step forward in optimizing the post-operative management of patients undergoing knee prosthetic surgeries. These integrative solutions not only improve the effectiveness of rehabilitation but also enhance communication between the patient and the care team, contributing to ensuring a faster, more efficient, and more personalized healing journey.

In addition to the implementation of mobile apps and wearables, advanced artificial intelligence systems are emerging that play a crucial role in the revolution of orthopedic surgery and post-operative rehabilitation. WalkAI is the artificial intelligence section present in myMobility in the ZBEdge ecosystem and is a significant example of how technology is contributing to improving the quality of care provided to patients undergoing knee prosthetic surgeries. One of its most relevant features is its ability to identify early deviations or delays in a patient’s rehabilitation. For example, if the system detects a decrease in the patient’s physical activity or slower-than-expected progress, it can alert the care team in real-time. This functionality allows caregivers to intervene promptly, adapting the rehabilitation plan or performing medical procedures based on the specific needs of the patient. This way, potential complications or delays in recovery are avoided, ensuring that the rehabilitation path remains highly efficient [14,23].

These systems not only improve the quality of care provided but also offer greater peace of mind to patients, knowing that they are constantly monitored and supported during their healing journey.

An example of this trend is the myMobility app, which integrates with the ROSA^®^ (robotic surgical assistant) robot used in the operating room (Zimmer Biomet, Warsav, IN, USA).

The myMobility^®^ orthopedic care management system (Zimmer Biomet, Warsaw, IN, USA) is a platform designed to provide personalized surgical experiences to qualified patients. It facilitates the creation of procedure-specific telehealth protocols, monitors patient engagement, tracks activity levels, and ensures compliance throughout the surgical journey [10,11,23,24].

The myMobility^®^ platform is an application (*App*) available for iOS (iOS is a trademark or registered trademark of Cisco in the USA and other countries and is used under license by Apple, Inc., Cupertino, CA, USA) and Android™ devices.

The aim of this study is to explore the objectives, functionalities, and outcomes of myMobility^®^, along with the associated problems and opportunities. Applying the *Wald Criteria* as previously described by Lonner et al. [23,25], we additionally propose strategies to improve patient recruitment, enhance adherence, and create a comprehensive patient journey for optimized surgical experiences, assuming that the involvement of various stakeholders, including surgeons, patients, physiotherapists, caregivers, administrative assistants, staff, and developers, is crucial for the successful implementation of the platform.

## 2. Materials and Methods

The analyzed patients were prescribed the use of the care management platform and underwent primary unilateral or bilateral total knee arthroplasty. The study spanned from January 2021 to February 2023 and specifically employed the ROSA^®^ Knee System (Zimmer Biomet, Warsaw, IN, USA) [26,27,28,29]. Patient data included records from three surgeons, ensuring a diverse and comprehensive dataset for analysis. This study obtained institutional review board approval and a subsequent waiver of consent and authorization (IRB NK5022). Each participant in this study underwent robotic-assisted total knee arthroplasty (raTKA) through a collaborative robotic system, as previously described [21]. The study’s structure extended through different phases of the care management platform, including consent, registration, activation, and utilization of pre- and post-operative rehabilitation programs. A survey was meticulously created and conducted to obtain information from patients actively using the app.

### 2.1. Selection

The survey was sent only to active patients on myMobility who underwent primary total knee replacement at a variable distance from the date of surgery, including patients undergoing bilateral arthroplasty. The selection criteria further refined the pool to include only patients operated on by the three most experienced surgeons, excluding those who underwent revisions of the primary prosthesis. The verification process involved searching for the names of the selected patients from myMobility in the hospital’s clinical data collection system. This was necessary as myMobility is currently not designed for patients undergoing revision procedures for joint replacement. Patient eligibility for participation was based on factors such as age (18 or above), fluency in the Italian language, and the ability to provide informed consent. The uniformity of the rehabilitation treatment protocol was maintained across all patients, allowing for consistent monitoring of progress by surgeons and physical therapists through a dedicated website.

### 2.2. Enrolling

All patients undergoing robotic-assisted surgery or, in general, joint replacement (including total hip arthroplasty and unicompartimental knee arthroplasty) at our center had previously signed an informed consent covering the use of personal data, also aimed at recording data on myMobility and the ZBEdge ecosystem (Zimmer, Warsaw, IN, USA), and the use of direct messaging. Through the filter labeled “active” on the myMobility app, it was possible to quickly identify patients actually using the app or some functions of it during post-operative rehabilitation or even further. A manual check made it possible to select patients undergoing total knee arthroplasty to whom the direct message containing the external link for the survey was sent in order to filter out potential responses from identical IP addresses or those originating from implausible IP addresses. Patients were contacted through the messaging service provided by myMobility. To eliminate potential biases, the survey coordinator was distinct from the surgeons performing the surgeries. Contacts were performed only through the myMobility app to respect patients’ privacy and not to obtain further unnecessary personal data. Patients received a unique external link to access the questionnaire anonymously, ensuring that only those who received the link could complete the survey directly from their personal device. An additional layer of verification occurred through an IP address cross-check, confirming the geographical origin of the responses.

### 2.3. Survey Implementation

The survey instrument was meticulously crafted by the principal authors of the study and comprised two sections. The first, consisting of 9 questions, particularly related to the technical aspect of the app; the second, consisting of 10 questions, explored general satisfaction and aimed at identifying the behavior and preferences of the patients. The questionnaire was intentionally designed to refrain from collecting any confidential health or personal information, thereby obviating the need for further consent. Most questions included mutually exclusive dichotomous answers (“yes” or “no”) with an additional option (“not sure”) to ensure clear, direct, and comparable answers. There were also an open-ended question and two multiple-choice questions (relating to the function judged most useful, the device used, and any suggestions to improve the app) to provide an analysis as complete as possible. The questionnaire was intentionally designed with a limited number of questions, each formulated in a clear and concise manner. This approach aims to prevent patients from losing interest during the completion process. By employing straightforward language and minimizing textual content, the intent was to ensure that respondents could easily navigate through the survey, maintaining their attention and engagement. The emphasis on brevity and simplicity was a deliberate strategy to enhance the overall effectiveness of the study, encouraging a higher response rate and valuable input from participants.

### 2.4. Data Analysis

The questions were hosted on the online service surveymonkey.com, which automatically provided a percentage analysis and the absolute frequencies of the answers obtained. A premium institutional account was created on the website with the single purpose of obtaining the requested information for this research. Surveymonkey’s automated indexing system streamlined the data analysis process, with results conveniently compiled into an .xls file and consequently converted into graphical illustrations. Demographic and statistical analyses ensued, encompassing mean age and gender. A 95% confidence interval was applied, leveraging the Student’s t-score and z-score for accurate comparison and robust conclusions. Importantly, this study was conducted without external funding, ensuring the integrity of the research process.

## 3. Results

### 3.1. Demographic Analysis

As of the present date, our records indicate that 536 patients have registered on myMobility^®^ and have undergone robotic total knee arthroplasty (rTKA). The mean age of these registered patients stands at 68.6 years. The gender distribution shows that 45.1% are male and 54.9% are female. The interviewed patients were, on average, 485 days post-intervention, with a standard deviation of 187.5.

Regarding the active patients using myMobility^®^ at the time of the survey, their demographic breakdown was as follows:

- Total count: 218 patients;
- Gender distribution: 56.5% male and 43.5% female;
- Average age: 63.45 years;
- Standard deviation: 9.3 years.

During the survey period, the demography of active patients on myMobility included a total of 218 individuals. The gender distribution among these active patients was 56.5% male and 43.5% female. The average age of this group was 63.45 years, with a standard deviation of 9.3 years. While the mean age for men was marginally lower than for women (62.4 versus 64.9), this difference did not reach statistical significance (t ≈ 1.39). However, a statistically significant age difference was observed between the active population on myMobility and the overall registered patient population (t ≈ 5.94). Finally, the M/F ratio did not exhibit a significant difference between the two groups (z ≈ −1.43).

No additional demographic data were collected from the respondents. All demographics are resumed in Table 1.

### 3.2. Response Rate Insights

**Figure 1:**
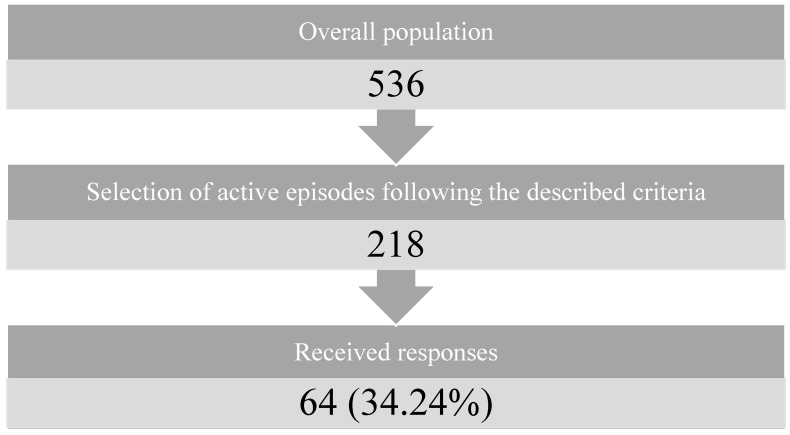
Flowchart of the workflow of the study.

Out of a total of 217 active patients (accounting for 218 procedures due to a bilateral total knee replacement) who had undergone primary total knee replacement, 64 responses were received, constituting a response rate of 34.24%. Each respondent diligently completed both questionnaires, a fact confirmed through IP address cross-checking. The survey spanned a period of two months, starting on 29 April 2023 and concluding on 29 June 2023, although the majority of responses were received within the first two weeks, with a solitary response arriving six weeks later (Figure 1).

### 3.3. Survey Results and Analysis

The first survey, focused on the technical aspects of the myMobility app, unveiled an overwhelmingly positive response from participants. A remarkable 100% of respondents found myMobility easy to use from the registration phase onward. A mere 13% of participants (8 patients) required assistance from the toll-free number for technical support. Furthermore, 93.3% reported feeling more informed since using myMobility and expressed ease in finding the information they sought. The rest answered “not sure” to these two questions. The primary devices for accessing myMobility were smartphones for 93.3% of respondents, with the remaining users opting for tablets and none using PCs. Notably, 37.5% of patients (24 patients) also owned a smartwatch.

In terms of functionality, the Education feature emerged as the most valued, garnering 75% of votes, followed by Routine (18.75%) and Statistics (6.25%). Interestingly, no patient identified the Messages and Rating features as the most important. When asked for suggestions or desired changes, all 64 respondents remained silent, offering no specific recommendations.

The second survey, the one relating to general patient satisfaction, provided further insights. Virtually all of the respondents expressed a willingness to recommend the use of myMobility, with only one patient expressing uncertainty, answering “not sure”. A notable 87.5% (56 patients) claimed success in achieving rehabilitation program goals, while the remaining respondents (8 patients) answered in the negative. Similarly, 87.5% (56 patients) deemed myMobility crucial in their rehabilitation process, and the remaining 8 patients answered “not sure”. Nearly all respondents (60 patients, 93.3%) reported a reduction in surgical anxiety through myMobility usage. The remaining four patients answered “not sure”. While 100% of participants appreciated the care team’s ability to monitor their progress and found exercise reference videos helpful, 56.25% (36 patients) expressed uncertainties about the promised faster contact and increased surgeon attention, answering “not sure” to the related questions.

A noteworthy finding was that 21.9% of patients had not engaged a physiotherapist, relying solely on myMobility for rehabilitation. Additionally, a slight majority (34 patients, 53.1%) expressed support for introducing a function to compare their own progress with other users, while 8 users (12.5%) were opposed, and the remainder were uncertain, answering “not sure”.

**Figure 2:**
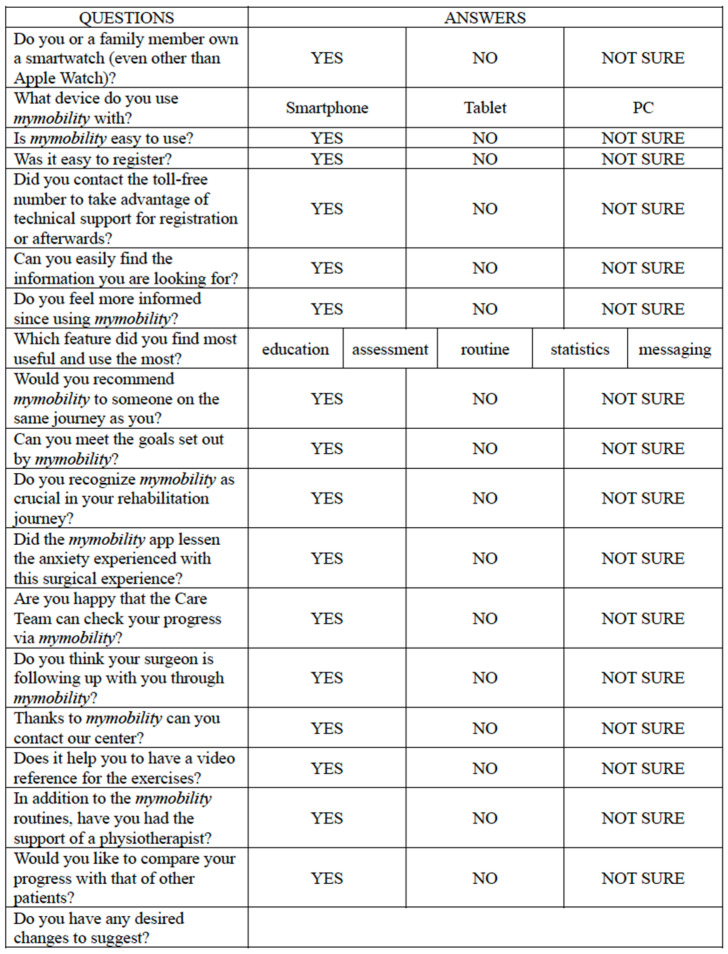
Text of the survey.

**Figure 3:**
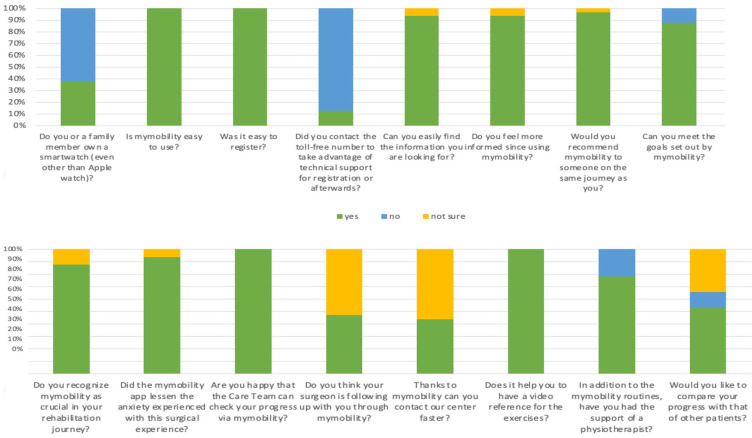
Graphic representation of the output of the administered survey.

**Figure 4:**
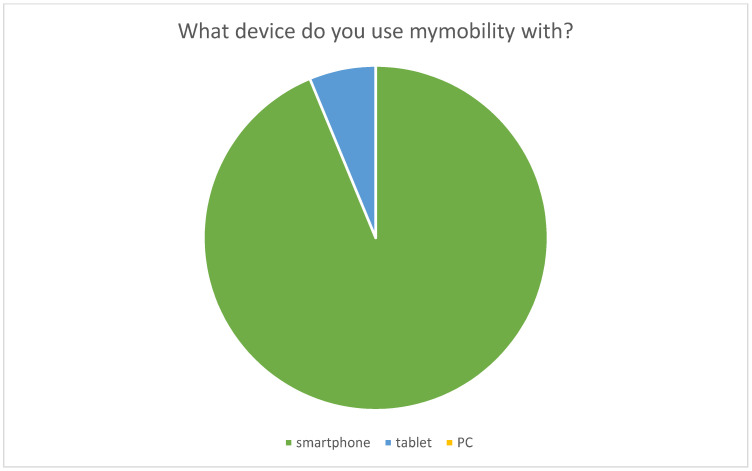
Graphic representation of the output of the administered survey.

**Figure 5:**
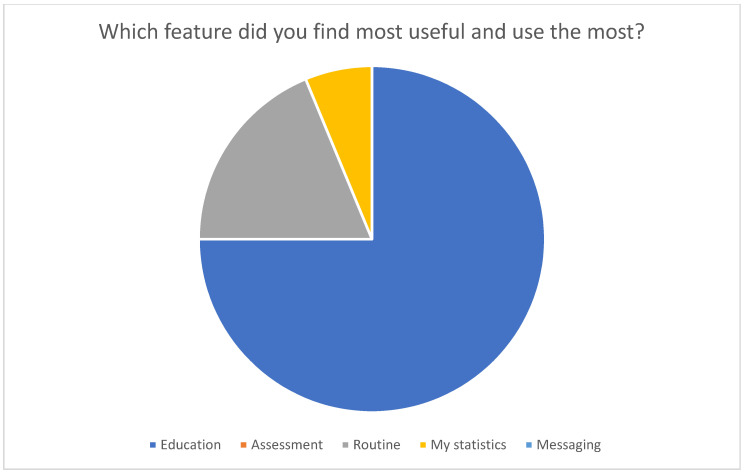
Graphic representation of the output of the administered survey.

The text of the survey and its results are graphically represented in Figure 2, Figure 3, Figure 4 and Figure 5.

## 4. Discussion

The initial findings of this study reveal that an analysis of the app’s usage and survey responses highlights several key points. Despite a favorable initial consent rate (>70%) for app usage, the number of patients actually activating the app decreases to 15–20%, with only around 10% using it correctly. Moreover, the percentage of patients utilizing the app to its maximum potential drops to less than 5%. This survey has brought forth varied results that necessitate evaluation and analysis.

Patients who participated in the survey generally expressed a positive view of the app, finding it highly valuable for educational purposes. However, they did not feel that the medical staff actively engaged with them through the app, nor did they perceive the platform as enhancing their communication with the hospital. Additionally, a majority of surveyed patients demonstrated a keen interest in a solution that incorporates a comparative analysis of their rehabilitation progress within the application, juxtaposed against the advancements of their peers. This inclination towards this kind of feature underscores the importance of a sense of achievement and motivation in a competitive yet supportive environment, which could ideally enhance patients’ engagement and adherence to rehabilitation protocols.

These results present opportunities for improving the utilization of these platforms during the post-operative phase. Applying the Wald Criteria, as described by Lonner et al. [19], entails examining the results from a distinct perspective and adopting a different perception of the problem. This approach aims to emphasize the opportunities arising from these results, facilitating the identification of solutions to enhance the outcomes of the current situation.

The analysis involves three main phases: patient recruitment, adherence, and experience. Crucially, it is evident that involving various stakeholders is essential for successful platform implementation. The roles and responsibilities of surgeons, patients/caregivers, staff, and company developers must be clearly defined and specified. Effective communication and collaboration among stakeholders throughout the surgical journey are necessary.

### 4.1. Enhancing Patient Recruitment

As of today, there are no operational guidelines for implementing telerehabilitation in the orthopedic scenario [24,25]. To bolster patient recruitment, adjustments to current processes are recommended. A suggested strategy involves eliminating pre-selection criteria from the recruitment protocol and enrolling all joint replacement patients regardless of instruction level, age, or device access [24]. Non-adhering individuals should be excluded subsequently. The platform should be integrated into the standard patient pathway, supported by strategies like informative leaflets and registration in pre-hospitalization checklists [26]. Conducting pre-operative educational programs or outreach initiatives to inform the patients about the surgery could include a section about the use and advantages of this solution. In this view, it is important to create a collaborative environment between healthcare professionals and tech assistants. Highlighting the app’s ability to tailor rehabilitation plans to individual needs can also be a winning strategy and can potentially attract patients.

### 4.2. Improving Adherence

Improving adherence is crucial for the app’s success. Strategies should align with the patient preferences identified in the survey. Providing comprehensive pre-operative information through video tutorials [27], integrating informed consent processes [28], and enabling direct patient-staff communication are pivotal for this phase [29]. Tailoring the platform to individual patient needs emerges as a key consideration [30]. In a more specific way, some tips about it could include a better user-friendly design with a straightforward interface and easy navigation and the implementation of accessibility features within the app to accommodate users with various abilities, including features such as voice commands, adjustable font size, or compatibility with screen readers. Moreover, the application already offers multiple languages, but it is important to add even more of them in order to overcome potential language barriers.

### 4.3. Creating an Inclusive Patient Journey

For optimized surgical experiences, establishing a comprehensive patient journey within the myMobility^®^ system is proposed. This involves incorporating rapid recovery protocols and AI integration, such as chatbots, to enhance patient experiences and provide immediate support [31]. Building a platform community through avatars, competition elements, and rewarding systems should also be discussed, along with providing features like forums, discussion boards, or virtual support groups where users can share their experiences and create a supportive environment with gamification elements.

In this regard, Pariser et al. have suggested that minimal interventions such as providing written information, setting goals, and creating action plans can result in improvements in terms of depression and pain among patients with osteoarthritis [32]. Feedback integration is another feature that could contribute to creating a sense of ownership and participation and giving useful advice to the developers.

In the near future, artificial intelligence (AI) and deep learning (DL) can significantly enhance chatbots and predictive models to improve the inclusive patient journey through surgery and rehabilitation. Chatbots driven by AI can engage with patients with natural language and learn from interactions, adapting responses based on patients’ behavior. Algorithms can predict the intensity levels of exercises according to individual capabilities and recovery rates, enabling timely adjustments to rehabilitation plans as well. To our knowledge, patient management applications are limited as of today. MyMobility^®^ is the only one that now offers a section of AI able to predict the speed of walking 90 days after surgery in the immediate post-operative time. Further improvements in the use of AI should be discussed.

### 4.4. Key Performance Indicators (KPIs)

Establishing KPIs is critical for assessing MyMobility^®^’s effectiveness. Specific KPIs for monitoring the platform’s success must be individualized. They should include patients’ engagement metrics (measuring the number of active users over a specific period and session duration), improving the effectiveness of rehabilitation, patients’ satisfaction (collecting patients’ feedback and reviews and identifying areas for improvement), accessibility and inclusivity (analyzing usage patterns across different demographics and monitoring interactions with support services), technical performances (tracking technical aspects, such as loading time or any reported crashes or glitches, and assessing the app’s compatibility with various devices and operating systems), improving the evaluation of adherence to rehabilitation plans and health metrics (such as pain reduction, improved range of motion, and enhanced functionality), data security and privacy, and correct usage. Regular surveys are emphasized for tracking patients’ satisfaction and gathering feedback.

### 4.5. Future Perspectives

The promotion of mHealth and tele-rehabilitation should be advocated, as they are now recognized to provide benefits for patients in terms of both economic considerations and time efficiency [10,33,34]. Moreover, they have been shown to be non-inferior and, in certain instances, even superior in terms of outcomes [35,36,37,38]. Furthermore, considering the unanimous contribution of the app in reducing pre-operative anxiety, it is crucial to emphasize that its role in influencing post-operative clinical outcomes remains a topic of debate [39,40]. This aspect should not be underestimated in patient management.

The accurate collection of intra-operative data through robotic systems offers valuable insights into joint kinematics, driving innovations in surgical software for reproducible and tailored outcomes. Artificial intelligence and machine learning algorithms show promise in predicting functional outcomes, including mobility data. However, the integration of intra-operative data into predictive models for total knee arthroplasty remains an unexplored area.

In the realm of patient management, contemporary platforms play a pivotal role within broader ecosystems where data are stored and seamlessly integrated. These ecosystems serve as fertile ground for the intersection of artificial intelligence (AI) and deep learning (DL), revolutionizing surgical decision making. The principles laid out by Wald underscore the need for a nuanced approach when applying traditional outcome measures to robotic interventions, especially knee prosthetics.

The advent of TKA within robotic frameworks challenges conventional outcome metrics. Unlike traditional assessments, the incorporation of AI and DL demands a reevaluation of success indicators. Metrics such as patient satisfaction emerge as critical benchmarks, reflecting the evolving landscape of orthopedic interventions. The very stimulus propelling the development of platforms tailored for patient management lies in the disruptive influence of robotics.

Robotic systems, embedded within these comprehensive data ecosystems, leverage AI and DL algorithms to navigate the intricate nuances of surgical decision making. These technologies not only enhance procedural precision but also contribute significantly to the patient’s overall experience. The dynamic shift from conventional metrics to patient-centric evaluations aligns with the essence of Wald’s principles, emphasizing the need for a customized approach in the era of robotic orthopedics.

The advantages of embracing robotic technologies extend beyond the operating room, permeating into postoperative phases. The emphasis on patient satisfaction as a primary outcome underscores a paradigm shift in assessing the success of TKA interventions. As these platforms continue to evolve, their integration into larger healthcare ecosystems heralds a new era where the symbiotic relationship between robotics, AI, and DL becomes indispensable for advancing surgical practices and optimizing patient outcomes.

Recommendations for further research and development should encompass evaluating long-term outcomes, integrating AI technologies, expanding the platform’s scope, and emphasizing continuous improvement to address evolving patient needs. In this view, physicians’ feedback is important as well. They should be interviewed about the clinical validity of these solutions and patient safety, identifying potential risks associated with the app’s use, and how they perceive the app’s role. Alignment with treatment plans should be assessed. Moreover, physicians can offer insights into patients’ compliance and engagement levels with the app and identify limitations and concerns. Data security and the right communications can be ensured by them. Finally, physicians’ feedback contributes to the app’s alignment with evidence-based practices, enhancing the credibility of the app within the medical community. Acknowledging the limitations of the study, which include secondary data analysis of anonymized data and a limited survey response within the studied population, the use of AI will be crucial in processing extensive data and influencing outcomes throughout the care journey.

### 4.6. Limitations

This study is subject to several constraints. Firstly, it exclusively involves patients treated by the three surgeons with the highest surgical case volumes. This may introduce a potential bias; however, it is partially mitigated by the fact that these patients constitute over 80 percent of the total population undergoing total knee replacement (TKR) procedures at the center.

A second limitation arises from the exclusive administration of the questionnaire via the mobile application. While this could be considered a partial limitation, it aligns with the study’s objective to assess the extent of app usage and patients’ actual access to it.

The third limitation pertains to the survey’s selective consideration of a subset of potential questions. However, the chosen questions were deliberate, aiming to elucidate issues and opportunities associated with app utilization.

Lastly, the decision to apply the Wald principles may be subject to scrutiny. Nevertheless, it represents an original and intriguing approach for the authors to addressing the problem at hand.

## 5. Conclusions

In conclusion, the myMobility^®^ system presents substantial potential for revolutionizing surgical care through the enhancement of patient engagement and outcomes. By addressing the identified challenges and implementing the proposed strategies, its utilization can be optimized, resulting in enhanced patient care and satisfaction.

The platform holds the capacity to reshape surgical experiences, elevating patient engagement, enabling remote monitoring, and facilitating personalized care. The integration of recruitment, adherence, and comprehensive patient journey strategies can further optimize its effectiveness.

## Figures and Tables

**Table 1:** Demographic data.

Total count of registered population	536 episodes	
Average age of registered population	68.6 years	
Gender distribution of registered population	45.1% male	54.9% female
Total count of active population on the platform	218 episodes	
Gender distribution of active population	56.5% male	43.5% female
Average age of active population	63.45 years	
Average age of active male population	62.4 years	
Average age of active female population	64.9 years	
Average time post-intervention	485 days	

## Data Availability

Associated data are available in a data repository.
